# Design and Fabrication of Microscale, Thin-Film Silicon Solid Immersion Lenses for Mid-Infrared Application

**DOI:** 10.3390/mi11030250

**Published:** 2020-02-27

**Authors:** Gil Ju Lee, Hyun Myung Kim, Young Min Song

**Affiliations:** School of Electrical Engineering and Computer Science, Gwangju Institute of Science and Technology, 123 Cheomdangwagi-ro, Buk-gu, Gwangju 61005, Korea; gjlee0414@gist.ac.kr (G.J.L.); kimhm3030@gmail.com (H.M.K.)

**Keywords:** micro-optics, microscopy, imaging systems, mid-infrared

## Abstract

Lens-based optical microscopes cannot resolve the sub-wavelength objects overpass diffraction limit. Recently, research on super-resolution imaging has been conducted to overcome this limitation in visible wavelength using solid immersion lenses. However, IR imaging, which is useful for chemical imaging, bio-imaging, and thermal imaging, has not been studied much in optical super-resolution by solid immersion lens owing to material limitations. Herein, we present the design and fabrication schemes of microscale silicon solid immersion lenses (µ-SIL) based on thin-film geometry for mid-infrared (MIR) applications. Compared with geometrical optics, a rigorous finite-difference time-domain (FDTD) calculation of proposed silicon microlenses at MIR wavelengths shows that the outstanding short focal lengths result in enhanced magnification, which allows resolving objects beyond the diffraction limit. In addition, the theoretical analyses evaluate the influences of various structural parameters, such as radius of curvature (RoC), refractive index, and substrate thickness, in µ-SIL. In particular, the high refractive index of µ-SIL is beneficial to implement the outstanding near-field focusing, which corresponds to a high numerical aperture. On the basis of this theoretical background, novel methods are developed for the fabrication of a printable, thin-film silicon microlens array and its integration with a specimen substrate. From the result, we provide a physical understanding of near-field focusing phenomena and offer a promising tool for super-resolution far-field imaging in the MIR range.

## 1. Introduction

As the resolution of a standard lens-based optical microscope imaging system is limited by diffraction to about half of the illumination wavelength, overcoming this resolution limit has been one of the primary interests in many areas of scientific and engineering research, such as nanophotonics, biochemical imaging, and material science [1,2,3,4]. In recent years, diverse studies of numerical calculations and experimental results have reported that far-field imaging beyond the optical diffraction limit can be achieved in real time only through the use of a conventional optical microscope with a wavelength-scale solid immersion lens (SIL) or a dielectric microsphere in close contact with the specimen of interest [5,6,7,8,9,10]. While most of the previous literature on this novel imaging scheme has focused on the visible wavelength band, no attempts have been made to investigate this method for the infrared (IR) region, despite the great demand of high-resolution imaging at the infrared range.

IR imaging is widely used for a broad range of applications including thermal imaging, biochemical imaging, and basic scientific research [11,12,13]. Especially, imaging tools with a mid-infrared (MIR) range (λ ~3–20 µm) provide a rich insight into the bio-chemical composition of cells and tissues, but face poor spatial resolution owing to the diffraction limit of light of long wavelength and relatively larger pixel size than the visible sensor owing to the low sensitivity [14,15].

With the rapid progress of various imaging applications in the MIR wavelength region, we considered that it would be of great interest to investigate super-resolution imaging with a wavelength-scale SIL in the MIR band.

In this paper, we demonstrate numerical calculations for a proposed optical design for evaluation of the focal properties and resolving power with various optical parameters in the MIR band. We also propose a novel fabrication and integration scheme for a microscale silicon solid immersion lens (µ-SIL) in a thin-film array structure as a far-field super-resolution imaging lens in the MIR region.

## 2. Materials and Methods

**Figure 1:**
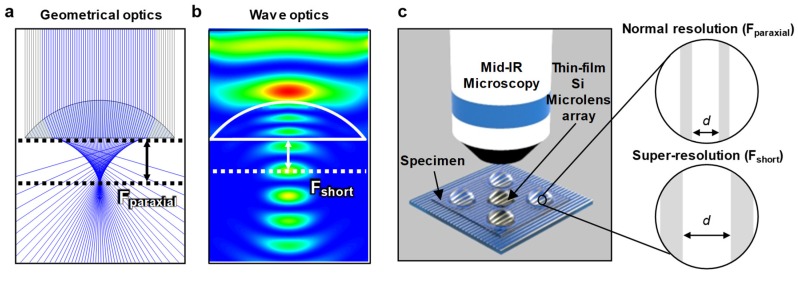
(**a**) Ray-tracing simulation and (**b**) wave optics simulation result. (**c**) (left) Schematic illustration of the imaging setup in which the microscale silicon solid immersion lens (µ-SIL) is placed on the specimen (not to scale). (right) The magnification difference is because of the short focal length.

When the optical lens size is much larger than the wavelength of light, the wave characteristics such as interference and diffraction can be ignored, and the optical phenomena can be expected by geometrical optics. Thus, optical imaging through lenses can be simply simulated by ray-tracing methods, as shown in Figure 1a. The paraxial focal length is described as *F_paraxial_* = *R*/(*n* − 1), where *R* is the radius of curvature (RoC). However, in wave optics, as the lens decreases to the wavelength, the interference between the incoming and refracted wave is more influential, resulting in a shorter focal length (i.e., *F_short_*) than *F_paraxial_* (Figure 1b). This shortened focal length in the SIL implies improved magnifying ability according to *M*1/*F,* where *M* is magnification, when the object distance smaller than the focal length. A schematic of an objective lens of a MIR-microscope system and a specimen substrate with µ-SIL shows that the image is enlarged by using µ-SIL as small as the wavelength with the focal length shorter than geometrical *F_paraxial_*, which has been mentioned in several previous articles for the visible wavelength range [5,6,7,8,9,16].

**Figure 2:**
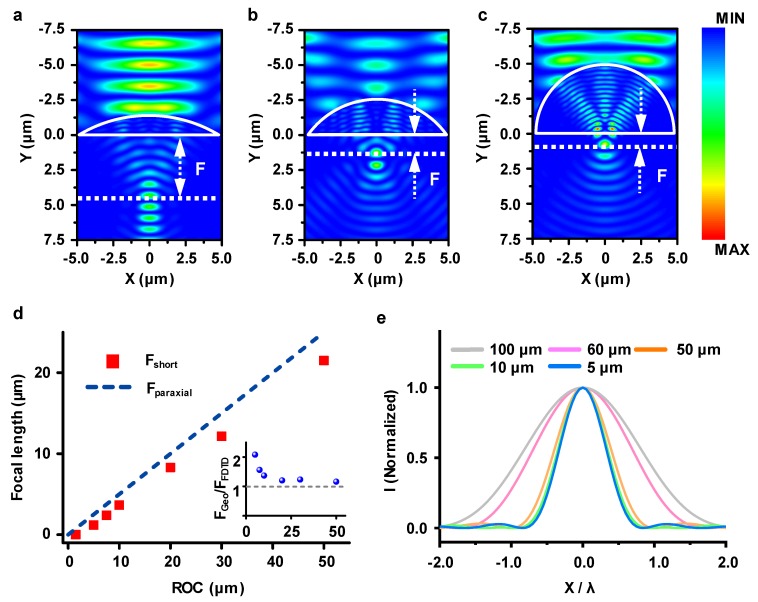
E-field intensity distribution of plane wave illumination onto a µ-SIL showing the variation of focal length for µ-SILs with radius to height ratios of (**a**) 3:1, (**b**) 2:1, and (**c**) 1:1 (hemispherical geometry). The refractive index used in this simulation was set as *n* = 3.5 (silicon). (**d**) Plot of focal length vs. µ-SIL radii of curvature (RoC). The line represents the calculated focal length using *F_paraxial_* = *RoC*/(*n*-1) and red markers are the results from the finite-difference time-domain (FDTD) simulation. The inset shows the ratio of geometrical focal length and those obtained by full width at half maximum (FWHM) simulation, respectively. (**e**) E-field intensity distribution on the focal region representing the variation of FWHM depending on the µ-SIL RoCs.

To study the optical performance of a microlens in the MIR range depending on various parameters (e.g., the dimension, RoC, and refractive index of materials used), we performed a series of finite-difference time-domain (FDTD) electromagnetic wave propagation simulations by Fullwave (RSoft, Synopsys, Mountain View, CA, USA) simulation tool [17,18]. As shown in Figure 2a–c, plane waves linearly polarized along the x axis were launched downwards along the y axis. The electric field distributions were calculated at λ = 3 µm wavelength by considering the lowest wavelength value of the MIR range (λ ~3–20 µm). It is noticeable that the back focal length of the µ-SIL shifts toward the rear vicinity of the lens as the RoC of the lens becomes smaller. From the perspective of practical fabrication, tuning the precise shape of microlens is quite challenging and fabrication of complete hemispherical microlenses of few micrometers is rather unachievable. In this regard, we set the ratio of the radius to the height of the microlens as 2:1 in the following calculations.

Figure 2d,e represent the focal length and full width at half maximum (FWHM) of the focal spot by which we could evaluate the spatial resolution of the combined microscope imaging system. As expected, Figure 2d shows the clear difference between the result of the calculated focal length from geometrical optics (blue-dashed line, *F_paraxial_* = *R*/(*n* − 1)) and those from the FDTD simulation (red markers). The obvious difference between them is because of a focal shift caused by diffraction of the incident light [19,20]. It should be noted that the degree of focal shift becomes clearer as the size of the lens scales down to the wavelength scale dimension, as shown in the inset of Figure 2d, representing that diffraction starts to dominate the focal property of the lens. For the same reason, as shown in Figure 2e, the FWHM of the focal spot becomes narrower as the dimension of the lens scales down and converges to ~0.5 µm (λ/6), at which we considered that the FWHM starts to become independent of the dimension of the lens. From the analysis of the simulation results, we confirmed that this transition occurs at RoC ~10 µm, clearly indicating the emergence of super-resolution imaging.

## 3. Results and Discussion

**Figure 3:**
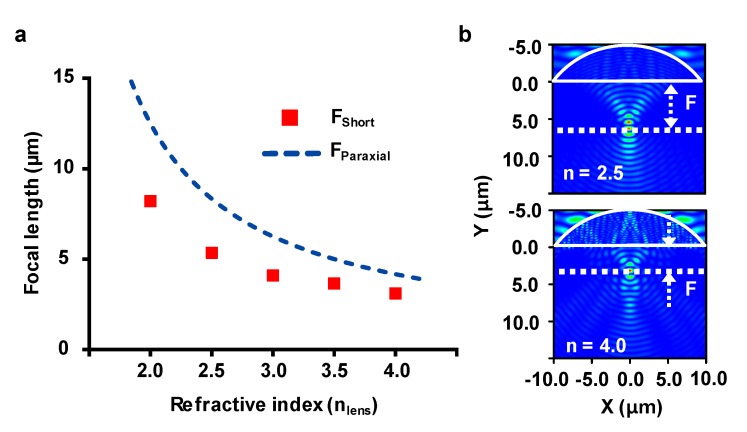
(**a**) Plot of focal length vs. µ-SIL refractive index. The line shows data calculated by geometrical optics and the red markers are the results from FDTD simulation. (**b**) E-field intensity distribution of plane wave illumination onto µ-SILs with different refractive indices, *n* = 2.5 (upper) and *n* = 4.0 (lower).

Figure 3a,b graphically show the effect of the refractive index of µ-SILs on the focal length. The dimension of the µ-SIL was set as 10 µm for the radius. A comparison between the geometrical optics calculation (blue-dashed line, *F* = *R*/(*n* − 1)) and the FDTD simulation results (red markers) shows the same trend over the change of refractive index with some degree of difference owing to the focal shift. Although a µ-SIL with a higher refractive index clearly shows a better focal property, one should note that most of the IR window materials, such as ZnS (*n* = 2.24), ZnSe (*n* = 2.43), Si (*n* = 3.48), and Ge (*n* = 4.0) [21], encounter severe Fresnel reflection at the interface, occurring as the higher refractive index difference may deteriorate the optical contrast of the resultant image.

**Figure 4:**
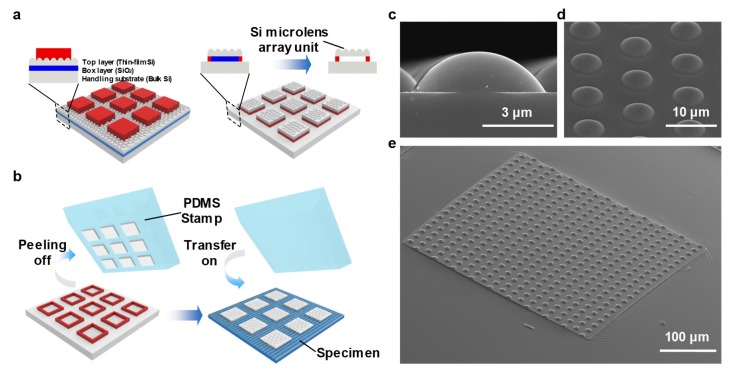
(**a**) Schematic illustration of the steps for fabricating printable silicon µ-SILs from a silicon on insulator (SOI) wafer and (**b**) subsequent transfer printing onto a specimen substrate. (**c**) Tilted-angle view of a scanning electron microscopy (SEM) image for a fabricated sample of printable silicon µ-SILs on rectangular silicon islands. (**d**) Magnified image of (c). (**e**) Cross-sectional view of a silicon µ-SIL unit.

Here, we propose a novel fabrication and integration schemes for a µ-SIL in a thin-film, microlens array structure. Despite the fact that most previous research has adopted the microsphere-type SIL, we consider a plano-convex microlens on a thin-film plate as the optimized design for µ-SIL-based MIR microscope systems for three main reasons: (1) the flat rear surface of the µ-SIL allows it be aligned and in contact with a sample much simpler than in the case of a microsphere; (2) the µ-SIL is fabricated as a transferable thin-film form factor, so that it can be reusable through a transfer printing process; and (3) engineering the optical properties of the lens can be realized relatively easily by use of the following fabrication processes. The target device size of wavelength scale could easily be implemented by well-known micro-fabrication technologies, such as photolithography, thermal reflow, and dry etching processes, which were employed for the fabrication of silicon microlens array [22]. In order to fabricate the µ-SIL, AZ5214 photoresist was spun and patterned by conventional photolithography on a silicon on insulator (SOI) wafer. Then, a thermal reflow process was conducted at 180 °C for 90 s. The underlying top Si layer was etched by reactive ion etching in an SF_6_/O_2_ gas mixture under optimized conditions. Precise control of the fabrication condition is needed to realize the desired shapes of the µ-SILs. To integrate the µ-SIL onto the specimen substrate of interest, we adopted and adjusted a series of micro-fabrication processes including transfer printing from the previous literature of flexible and stretchable devices, as illustrated in Figure 4a,b [23,24,25]. Therefore, as shown in Table 1, the thin-film silicon microlens array has a printable characteristic that cannot be achieved by the existing micro-fabrication technology [26,27,28,29,30,31,32,33,34], and thus has great advantages for biochemical imaging of cell/tissues compositions requiring alignment with uneven samples in mid-infrared wavelength. Scanning electron microscopy (SEM) images of the resultant µ-SILs at various magnifications are shown in Figure 4c–e.

**Figure 5:**
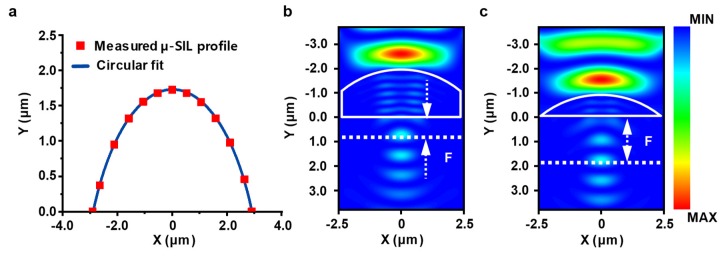
(**a**) The measured silicon µ-SIL profile with circular fitting (radius = 3.3 µm). (**b,c**) E-field simulations for two types of silicon µ-SIL structures: (**b**) with 1 µm thick silicon and (**c**) without the substrate.

The silicon µ-SIL manufactured by the above method extracted meaningful lens information through image analysis by a commercial tool (MATLAB, Mathworks, Inc., Natick, MA, USA). As shown in Figure 5a, as the profile of the fabricated µ-SIL matches the circular fitting, the lens was demonstrated in a nearly perfect plano-spherical convex structure. The geometrical information of µ-SIL is shown in Table 1, with a radius of 3.3 µm, a diameter of 5.8 µm, and a height of 1.723 µm. Further, the radius-to-height ratio (~1.9:1) is analogous to the prediction result used in the FDTD simulation. In addition, computation studies for the fabricated µ-SIL with and without the thin-film substrate are exhibited in Figure 5b,c. These simulations demonstrate that front focal lengths of µ-SIL are independent to the presence of the thin-film substrate. The independency of µ-SIL for the thickness of substrate allows for practical design of silicon µ-SIL, which facilitates fabrication and transfer-printing. Also, such an imaging feature of µ-SIL can control the distance between a sample and the fabricated silicon µ-SIL by modifying the thickness of thin-film substrate because the lens is conformally in contact with the sample using the transfer-printing method. This distance-variable property allows for the magnification adjustment of the obtained image [5].

**Figure 6:**
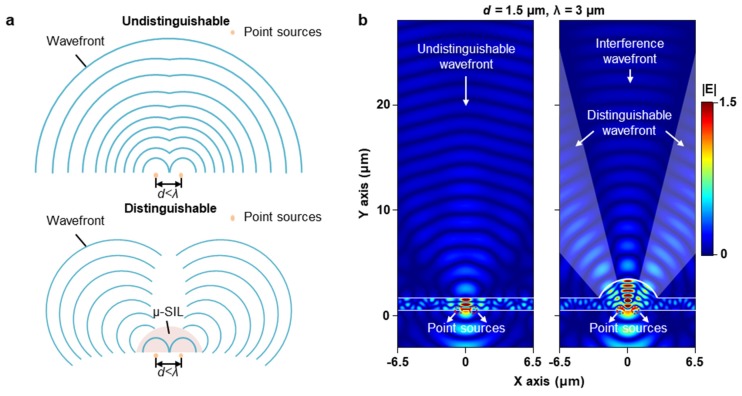
(**a**) Schematic illustration for super-resolution imaging by silicon µ-SIL. Without the silicon µ-SIL, closely-located two point sources (*d* < λ) are undistinguishable owing to the diffraction limit. However, the silicon µ-SIL can overcome the diffraction limit. (**b**) Electric field simulations using two point sources at *d* = 1.5 µm (left) without and (right) with the silicon µ-SIL.

The optical properties of silicon µ-SIL are illustrated in Figure 6a [5]. A wavefront of far-field is not separated when two point sources are located at a shorter distance than the wavelength (Figure 6a; undistinguishable). However, the silicon µ-SIL enhances the spatial resolution by remarkably high numerical aperture, resulting from the high refractive index of silicon (Figure 6b; distinguishable). Figure 6b computationally demonstrates the super-resolution of silicon µ-SIL. The simulation model of silicon µ-SIL has geometrical parameters listed in Table 2. Without the silicon µ-SIL, two point sources with *d* = 1.5 µm (i.e., *d* = λ/2) generate the undistinguishable wavefront in far-field. However, by introducing the silicon µ-SIL, two point sources could be classified even at far-field. Between two distinguishable wavefronts, the interfered wavefront is also observed.

## 4. Conclusions

In summary, we performed a numerical simulation to investigate the focal properties of µ-SILs in the MIR band, analyzing the dependence of the shape, RoC, and refractive index as a promising tool for far-field imaging beyond the optical diffraction limit. Further numerical analysis, such as electric dipole simulation for rigorous evaluation of the resolution enhancement, should be performed as a future work [35]. We also presented a fabrication method and integration methods for a printable µ-SIL-based MIR microscope imaging system. The computational results verify the applicability of silicon µ-SIL for super-resolution imaging. We believe that the proposed optical device could be a promising candidate for super-resolution far-field imaging systems for various MIR applications.

## Figures and Tables

**Table 1:** Information of microlens arrays for infrared optical applications.

Materials	Lens Shape	Deformability	Mechanism	Ref.
As_2+_Se_3_	Plano-convex	Rigid	Diamond turning and glass molding	[26]
Si	Plano-convex	Rigid	Photolithoraphy and dry etching	[27]
Si	Plano-concave	Rigid	Fs laser and wet etching	[28]
Si	Plano-concave	Rigid	Diamond turning	[29]
Si	Plano-convex	Rigid	Photolithoraphy and dry etching	[30]
Si	Plano-convex	Rigid	Anodic dissolution process	[31]
Ge-Se-Sb chalcogenide	Plano-convex	Rigid	Photolithoraphy and thermal reflow	[32]
SiC, GaAs	Plano-concave	Rigid	Fs laser and wet etching	[33]
a-Si/H	Plano-convex	Rigid	Micro-fabrication and CVD	[34]

**Table 2:** Geometrical information of the fabricated microscale silicon solid immersion lens (µ-SIL).

Radius (µm)	Diameter (µm)	Height (µm)	Radius to Height Ratio
3.3	5.8	1.723	~1.9:1
